# Does Twice-weekly Cabergoline Improve Anthropometrical and Biochemical Profiles in Prediabetes? A Randomized Double-blind Clinical Trial Pilot Study

**Published:** 2015

**Authors:** Navid Saadat, Hadi Esmaily, Mohammad Abbasinazari, Maryam Tohidi, Jamshid Salamzadeh, Farzad Hadaegh, Maryam Tolabi, Maryam Kalantar- Hormozi, Maryam Dibaj

**Affiliations:** a *Research Institute for Endocrine Sciences, Shahid Beheshti University of Medical Sciences, Tehran, Iran.*; b *Department of Clinical Pharmacy, School of Pharmacy, Shahid Beheshti University of Medical Sciences, Tehran, Iran. *; c *Islamic Azad University of Pharmaceutical Sciences Branch, Tehran, Iran.*

**Keywords:** Cabergoline, Prediabetes, Anthropometric, IGT, IFG, Glucose metabolism

## Abstract

Dopaminergic signaling is one of the regulatory pathways being investigated for its implication in glucose metabolism. The aim of this study was to determine the effect of cabergoline on biochemical and anthropometric parameters in prediabetes stage (impaired fasting glucose and impaired glucose tolerance). In this double blind, placebo-controlled, pilot study, 27 prediabetic adults were randomized to receive 0.25-mg cabergoline twice weekly for two weeks, followed by 0.5 mg twice weekly for next 14 weeks (n = 13) or placebo (n = 14). All subjects were advised to follow a 500 kcal-deficit energy diet. Fasting plasma glucose (FPG), oral glucose tolerance, glycated hemoglobin (A1c), fasting, and 2-h insulin were measured at baseline and at 16-week follow-up. Homeostasis model assessment (HOMA) 2 was calculated to estimate steady-state beta-cell function, insulin sensitivity, and insulin resistance. Our results showed significant reductions in fasting (P = 0.004**)** and 2-h plasma glucose (P = 0.01) after treatment, and significant improvements in beta-cell function (P = 0.03) and insulin resistance (P = 0.04) in the cabergoline group. The trend of non-significant A1c changes was decreasing in the cabergoline group versus an increasing trend in the placebo group. All anthropometric parameters were similar between the two groups. Our results revealed that twice-weekly cabergoline could improve glucose metabolism in prediabetes stage. Larger studies of longer duration are warranted to investigate the effect of cabergoline in preventing progression of prediabetes to type 2 diabetes mellitus.

## Introduction

It is estimated that developing countries in Asia and in the Middle East will have the largest increase in the prevalence of diabetes by 2030(1) and the incidence of type 2 diabetes mellitus (T2DM) has doubled in recent years (2). Additionally, our ability to prevent or predict T2DM is limited. However, epidemiological studies have identified certain risk factors of T2DM, including prediabetes stage [impaired glucose tolerance (IGT) and impaired fasting glucose (IFG)], T2DM in close relatives, obesity, and Asian, Hispanic or African-American ethnicities (3), Preventive methods are focused on postponing the onset of T2DM, preventing vascular complications, and delaying the failure of beta cells.

For selected individuals [(age < 60 years, BMI ≥ 35 Kg/m^2^, and history of gestational diabetes (GD)] who have IFG, IGT, or glycated hemoglobin (A1c) of 5.7–6.4%, when lifestyle modifications fail to improve glycemic indices, pharmacological agents should be considered. Among all agents, more evidence is available for metformin (4). Younger and more obese adults and women with a history of GD benefit more from metformin (4,5). However, all tested pharmacological agents (including metformin) are less effective than lifestyle interventions (6,7). Therefore, developing agents that have preventive effect on progression of prediabetes to T2DM is highly desirable. 

Even after normalization of glucose levels in prediabetes stage, these patients still remain at higher risk for atherosclerotic cardiovascular complications. Therefore, preventive agents that not only improve glycemic control are desirable. Hence in the present study, we evaluated a dopamine agonist. 

Dopaminergic signaling is one of the regulatory pathways in the central nervous system (CNS), which is implicated in many neurological processes, including motivation, pleasure, cognition, learning, and modulation of neuroendocrine signaling as well as glucose and energy metabolisms mainly at centers in the medial basal hypothalamus. CNS controls gluconeogenesis by sympathetic pathways, and other hormonal signals such as insulin, leptin, resistin, ghrelin, and glucagon-like pepide-1 (GLP-1) (8). 

Five cloned receptors have been identified to be involved in dopamine signaling. The D1 and D5 receptors are members of the D1-like family, whereas the D2, D3, and D4 receptors are classified as D2-like family. These receptors belong to the family of seven trans-membrane domain G protein-coupled receptors. Some evidence suggests the existence of D6 and D7 receptors (9). D1 and D2 receptors are 10–100 times more abundant than the D3, D4, and D5 subtypes (10). 

The effects of dopamine pathways on glucose and energy homeostasis could be present in two distinct ways: as a direct effect of catecholamine and an indirect prolactin effect on this system.

*Literature review*

Here, we will discuss two related pathways of dopamine mechanism of action based on published studies: 

*Direct effects of dopamine on glucose and energy homeostasis*

There is evidence to prove a clear association between obesity and a decrease in the expression of D2 receptors in the brain of obese individuals (11). In addition, Cincotta *et al.* showed that bromocriptine could reduce fasting and postprandial glucose levels in non-diabetic obese subjects. Other investigators showed that bromocriptine could reduce fasting plasma glucose and mean plasma glucose in diabetic patients (12). In addition, medications that inhibit dopamine pathways, such as antipsychotic medications, could impair β-cell function and increase insulin resistance (13). 

After clinical trials confirmed the efficacy and safety of a rapid release formulation of bromocriptine (Cycloset®) in T2DM, it was approved in 2010 as an adjunct therapy for T2DM. Hence, bromocriptine was the first dopamine agonist approved to improve glycemic control in T2DM patients. Bromocriptine is an ergot derivative, which stimulates D2 receptor, inhibits D1 receptor (14), and has certain serotonergic properties such as partial agonistic effect on 5-HT2B receptors and inhibitory effect on 5-HT2A receptors (15).

*Hyperprolactinemia´s effects on glucose and energy homeostasis*

Prolactin receptors are present in the pituitary glands, liver, pancreas, adrenal gland, and skeletal muscle. Like insulin, when prolactin binds to its receptor, it causes it to dimerize with another prolactin receptor. This results in the activation of Janus kinase 2, a tyrosine kinase that initiates the JAK-STAT pathway. Studies in humans have linked hyperprolactinemia to metabolic syndrome, glucose intolerance, obesity, and insulin resistance (16,17). Other studies have shown the involvement of prolactin in islet *β*-cell growth, development, and differentiation and insulin and adiponectin pathways (16,18). 

Moreover, prolactin can regulate the production/secretion of insulin and adiponectin (16) and cause changes in insulin metabolism (19), Dopamine agonists were shown to induce weight loss in hyperprolactinemic patients after significant decrease in prolactin levels (18,19). Cintia M. and colleagues have reviewed all suggested mechanisms by which hyperprolactinemia could disturb metabolic homeostasis, such as increased leptin resistance, decreased adiponectin levels, and increased hypothalamic pressure (20), However, it is not known whether hyperprolactinemia induced these complications or the reduction in dopaminergic tone induced hyperprolactinemia and other metabolic complications.

Cabergoline is also an ergot derivative dopamine agonist with long lasting properties, it is the first choice among dopamine agonists in the treatment of hyperprolactinaemia because of its efficacy, ease of use and side effect profile (21). Cabergoline administered once or twice a week and has much less tendency to cause nausea than bromocriptine and it is superior to bromocriptine in decreasing the serum prolactin concentration (22,23). Cabergoline is also an ergot derivative dopamine agonist with long lasting properties. It is the first choice among dopamine agonists in the treatment of hyperprolactinemia because of its efficacy, ease of use, and side effect profile. Cabergoline is administered once or twice a week, has much less tendency to cause nausea than bromocriptine, and is superior to bromocriptine in decreasing the serum prolactin concentration. 

Effect of cabergoline has been studied on anthropometric parameters, lipid profile, and insulin resistance in hyperprolactinemic patients; but few studies have examined its effect on patients with normal prolactin levels. In addition, the effects of cabergoline on prediabetes patients have not been investigated. We investigated the role of dopamine/prolactin in progression of *β*-cell function impairment from prediabetes stage to T2DM and whether cabergoline could stop this progression. The aim of the present study is to determine if cabergoline is effective in alleviation of anthropometric and biochemical profiles in prediabetic patients.

## Experimental

*Participants*

We designed a randomized, double blind, and placebo-controlled clinical pilot study involving 27 prediabetic patients. The clinical study was registered in the Australian and New Zealand Clinical Trials Registry with registration code of ACTRN12613001121752.

Ethics committee approval was obtained from the Shahid Beheshti University of Medical Sciences before starting the study as per the provision of the Helsinki declaration (2000). Eligible patients were asked to sign consent forms for enrolment in the study.

Subjects were included in our study were women and men aged between 30-65 years, selected from volunteers involved in the Iranian National Diabetes Screening Program in 2013, which were invited for reassessing and voluntary involving in our randomized clinical study. Women were either infertile or used appropriate contraceptive measures other than oral contraceptives. Subjects were excluded for the following reasons: previous history of hypersensitivity reactions to cabergoline or other ergot derivatives; ongoing pregnancy or lactation; history of diabetes, valvular or fibrotic cardiovascular disease, lung disease, any psychiatric disease that required treatment with any medications that affect the dopamine pathway, uncontrolled thyroid disease; alcohol or substance abuse; current smoker or smoking cessation in last three months; history of renal and hepatic impairment; uncontrolled hypertension; bulimia or anorexia nervosa; weight loss ≥5 Kg within the past year; use of medications that could affect prolactin levels, body weight, glucose homeostasis, and lipid profile; use of weight loss medication for more than two weeks during the prior 180-day period; and administration of opiates or glucocorticoids in pharmacological dosage (>7.5 mg prednisolone or equivalents for ≥30 days in last year) within 30 days prior to our conformational screening. 

*Experimental details*

Eligible subjects were assigned to the placebo or active compound groups using a randomized table in the obesity and metabolic disorders clinic (pilot study algorithm). The active compound was cabergoline (0.25 mg) given orally twice a week before bedtime for two weeks, and then escalated to 0.5 mg biweekly for the next 14 weeks. Nutritionists from the Clinical Research Center provided a high fiber diet plan with a 500 kcal deficit calculated from the Harris Benedict formula adjusted for activity, with a maximal intake of 1800 kcal/day [For women, Basal metabolic Rate = 655 + (9.6 x weight in kilos) + (1.8 x height in cm) - (4.7 x age in years). For men: Basal metabolic Rate = 66 + (13.7 x weight in kilos) + (5 x height in cm) - (6.8 x age in years)]. Subjects were asked to maintain a stable physical activity level, and compliance to the medication and diet were assessed by telephonic follow-ups every week. At the initial and final visits, a 75-g anhydrous oral glucose tolerance test (OGTT) was performed with blood samples drawn at time 0 and 120 minutes. 

*Pilot study algorithm*

At the start and end of the study, height, weight, and waist circumference (WC) measurements were recorded; and pills were counted to check for adherence with study medication. Follow-up calls were made to monitor for adverse events and medication or lifestyle changes. Serum prolactin levels, which were expected to decline in subjects receiving active compound, were also indicative of subject compliance, but were measured at study completion to avoid “unblinding” the investigators.

Plasma insulin level and A1c were measured after completion. Homeostatic Model Assessment of Insulin (HOMA) 2 was calculated by University of Oxford HOMA2 calculator, and was represented as %B for steady state β-cell function, %S for insulin sensitivity, which was reported as percentage of normal population, and IR factor as an indicator of insulin resistance. 

*Biochemical assessments*

Serum samples were stored at −80 °C until assayed. Serum prolactin and insulin were measured by ECLIA analyzer, Cobas e-411 (Roche Diagnostics, GmbH, Mannheim, Germany), with intra- and inter-assay coefficients of variation of 1.7 and 1.8%, respectively for prolactin, and 0.8 and 1.8%, respectively for insulin. Serum glucose were measured by enzymatic colorimetry using Selectra 2, Vital Scientific Co. Dieren, Netherlands analyzer with Pars Azmun Co. Tehran, Iran kits, with intra- and inter-assay coefficients of variation of 0.6 and 0.7. A1c was measured by enzymatic method using Hitachi 911 chemistry analyzer (Roche Diagnostics, GmbH, Mannheim, Germany), with intra- and inter-assay coefficients of variation of 1.0 and 1.1, respectively.

*Statistical analysis*

The results are shown as mean ± SD. Data was analyzed using SPSS-15, and P < 0.05 was considered to be statistically significant. The primary endpoint was change in HOMA2-IR from baseline to week 16. This is a pilot study, and for more detailed information, this study should be repeated using a larger sample size based on this endpoint to detect difference between the two groups. Oxford university calculator was used to calculate HOMA2 parameters in this study (24). Results are presented as model estimated mean ± SEM for inter-group over time and intra-group at the same time comparisons based on model estimated 95% confidence intervals.

## Results

A total of 178 individuals who were considered as IFG/IGT subjects in the Iranian National Diabetes Screening in 2013, out of our primary telephonic survey of 178 subjects, 68 subjects met the primary criteria which were invited to reassessment, out of reassessed subjects, 27 participants were still in IFG/IGT stage, met the inclusion criteria, and consented to be randomized and involved in this study. A total of 4 subjects (14.81%) dropped out during the 16 week study period: 3 subjects from the placebo group (1 lost due to a foreign trip; 1 lost to follow up; 1 removed due to a fixed eruption which was may be a fixed drug eruption); 1 subjects from the cabergoline group (1 subject removed because of a resistant vertigo). Finally 23 participants completed the study (Figure 3, Alg.). There is no significant difference between two groups regarding baseline parameters such as either demographic (sex, age and BMI) or biochemical (A1c, FPG, 2 hours PG and Prolactin). Table 1 shows mentioned demographic and biochemical parameters in two groups. 

**Table 1:** Baseline demographic and biochemical parameters of studied patients

	**Placebo **	**Cabergoline **	**p-value **	**95% CI **
Sex (M/F)	4/7	7/5	0.29	
Age (y)	51.18 ± 2.81	56.66 ± 2.23	0.139	-12.89 — 1.92
BMI (Kg/m2)^a^	27.50 ± 1.21	30.67 ± 1.54	0.56	-7.29 — 0.98
FPG (mg/dl)^b^	102.90 ± 2.61	106.25 ± 2.33	0.35	-10.62 — 3.93
OGTT (mg/dl)	110.09 ± 11.41	143.25 ± 13.10	0.59	-69.59 — 3.27
A1c (%)^c^	5.70 ± 0.08	5.70 ± 0.13	0.99	-0.33 — 0.34
Prolactin (ng/mL)	9.65 ± 0.87	10.23 ± 1.86	0.09	-5.03 — 3.79

Values represent as mean ± SEM

a : Body Mass Index

b : Plasma glucose of 2 hour after OGTT

c : Glycated Hemoglobin A1c

**Figure 1:**
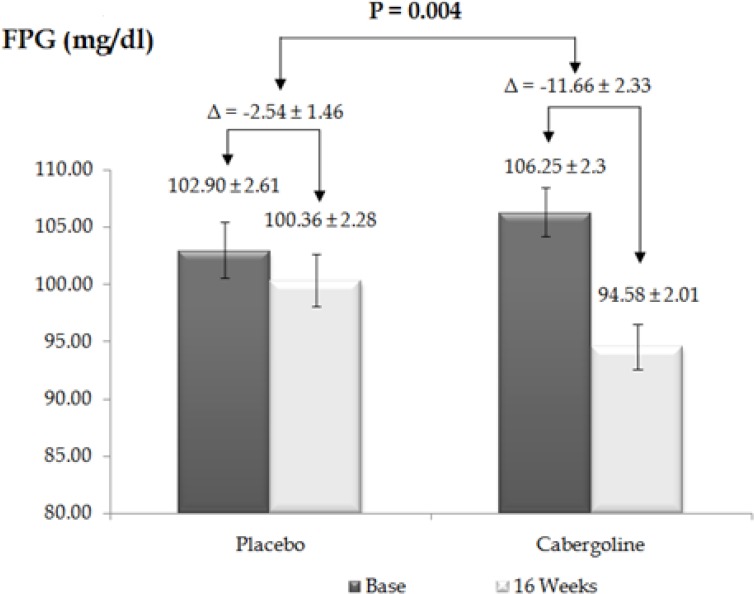
Fasting plasma glucose level in placebo and cabergoline groups before and after 16 weeks

**Figure 2:**
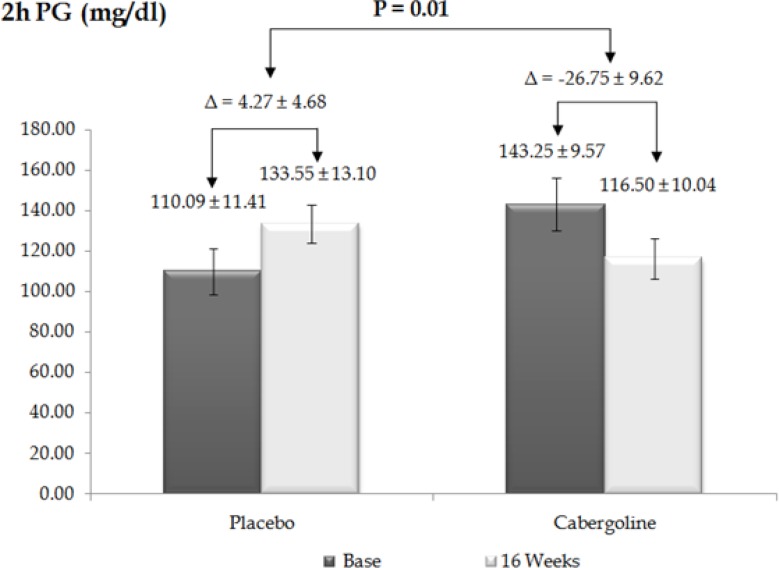
Post-prandial plasma glucose level in placebo and cabergoline groups before and after 16 weeks

Changes in anthropometrical and biochemical parameters over 16 weeks are presented in Table 2. As expected, prolactin levels significantly decreased in the treated group after four months of treatment (P < 0.001, 95% CI = 6.09–13.85). Statistically significant differences were observed between basal and 16 weeks post-treatment FPG levels (P = 0.004, 95% CI = 3.27—14.97) (Figure 1), and between the basal OGTT and after 16 weeks of treatment (P = 0.01, 95% CI = 8.1—53.94) between the two groups (Figure 2). 

**Figure 3:**
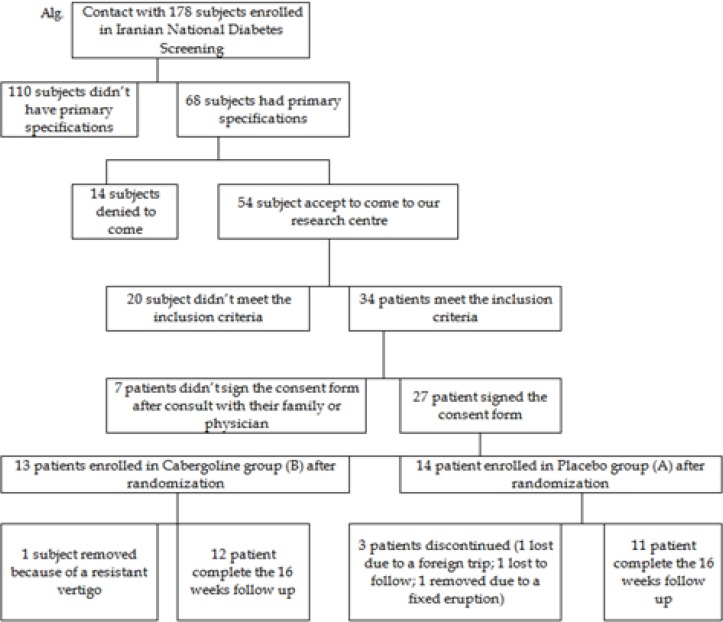
Flow of the study

As shown in Table 2, the trend of weight changes, WC, triglyceride, fasting insulin, and A1c was reducing in both groups over 16 weeks but the differences were not significant. After cabergoline treatment, HOMA2 %B was increasing, but the differences were not significant over 16 weeks of treatment. Statistically significant changes were observed in 2-h insulin levels (P = 0.02), HOMA2 %S (P = 0.03), and HOMA2 IR (P = 0.04).

**Table 2:** Comparing the anthropometric and biochemical parameters before and after 16 weeks treatment

	**Placebo**				**Cabergoline**			
	**Before**	**After 16 w**	**p-value**	**95% CI**	**Before**	**After 16 w**	**p-value**	**95% CI**
Sex (M/F)	4/10	4/7			7/6	7/5		
Prolactin (ng/mL)	9.65 ± 0.87	9.78 ± 1.01	0.92	-2.91 — 2.65	10.23 ± 1.86	0.29 ± 0.08	0.00	6.09 — 13.85
BMI (Kg/m2)	27.50 ± 1.21	26.83 ± 1.20	0.70	-2.98 — 4.35	30.67 ± 1.54	29.77 ± 1.42	0.67	-3.46 — 5.26
A1c (%)	5.70 ± 0.08	5.77 ± 0.10	0.64	-0.34 — 0.21	5.7 ± 0.13	5.59 ± 0.01	0.46	-0.20 — 0.43
Weight (Kg)	73.90 ± 4.35	72.18 ± 4.56	0.78	-11.43 — 14.89	81.41 ± 4.51	79.00 ± 4.16	0.69	-10.31 — 15.41
WC* (cm)	95.72 ± 3.5	92.36 ± 3.89	0.52	-7.56 — 14.29	104.75 ± 3.29	102.33 ± 3.44	0.61	-7.47 — 12.31
2h Insulin (µU/L)	66.22 ± 9.35	51.14 ± 10.48	0.30	N.NL.	77.32 ± 3.75	55.23 ± 7.79	0.02	2.84 — 4.34
HOMA2 %B	96.26 ± 17.36	80.90 ± 9.53	0.46	-27.30 — 58.02	85.63 ± 5.44	98.65 ± 6.06	0.12	-29.93 — 3.88
HOMA2 %S	101.16 ± 19.98	109.65 ± 22.91	0.78	-71.91 — 54.93	76.76 ± 7.24	98.65 ± 6.06	0.03	-41.40 — -2.28
HOMA2 IR	1.54 ± 0.33	1.40 ± 0.27	0.74	-0.76 — 1.05	1.46 ± 0.14	1.14 ± 0.04	0.04	0.00 — 0.64

Value represented as mean ± SEM

Bolded P values are statistically different before and after 16 weeks of treatment

Analysis done based on within group average changed over 16 weeks

* Waist Circumferences (WC)

## Discussion

Based on our litrature review on pubmed and scopus till Jan 2014, this is the first time that cabergoline was introduced as an agent to abate the progressive worsening of glycemic control from prediabetes to T2DM. Additionally, this is the first study that evaluates the effects of cabergoline on anthropometric and biochemical markers of glucose homeostasis in prediabetic patients who were not treated with any other hypoglycemic agents before. 

The first clinical study investigating the metabolic effects of dopamine agonists on glucose homeostasis was published in 1992; bromocriptine was the first agent tested in these studies. In 2010, the US Food and Drug Administration evaluated the results of bromocriptine rapid release formulation clinical trials, and approved it as an adjunct agent in addition to main oral agents for T2DM.

Some *in-vitro* investigations demonstrated the participation of D2R in insulin secretion. (25,26). In contrast, Roasti *et al*. showed that treatment of Parkinson’s disease patients with L-DOPA reduces insulin secretion upon OGTT (27), Kamath V *et al*., showed that bromocriptine did not change plasma insulin concentration in obese, non-diabetic, hyperinsulinemic women (28). Gibson *et al*. showed that cabergoline did not change fasting insulin in non-diabetic obese patients. Our results demonstrated that treatment with cabergoline decreased 2-h insulin level (P = 0.02), and showed a decreasing trend in fasting insulin level (P = 0.12) (29).

Proposed mechanisms by which dopamine agonists could improve glucose metabolism include gluconeogenesis suppression, induction of splanchnic glucose uptake and central effects. Gibson *et al.* showed that cabergoline did not change FPG and 2-h-PG in non-diabetic obese patients, with the average baseline FPG of 91.7 ± 2.7 mg/dL in the placebo group and 89.0 ± 2.7 mg/dL in the cabergoline group. In contrast, our results showed a dramatic decrease in FPG and 2-h-PG of patients with prediabetes, with an average baseline FPG of 102.90 ± 2.61 mg/dL in the placebo group and 106.25 ± 2.3 mg/dL in the cabergoline group. We hypothesize that this difference is because the patients included in the Gibson study had normal FPG levels. This difference suggests that the mechanism of cabergoline is not based on stimulation of insulin secretion but on increased insulin sensitivity. To investigate this finding we used Oxford HOMA2 calculator to estimate insulin sensitivity/resistance and β-cell function, and found that cabergoline could remarkably increase insulin sensitivity (P = 0.03), and decrease insulin resistance (P = 0.04). Our data also showed an increasing trend in *β*-cell function but it was not significant over four months (P = 0.12).

Our patients showed no significant changes in anthropometric parameters over four months of treatment. Also, the trend of changes was decreasing in both groups (−1.72 ± 0.86 Kg in the placebo group and −2.41 ± 1.28 Kg in the cabergoline group; P = 0.66). In contrast, several studies have demonstrated weight loss with the use of dopamine agonists. However, many of these studies included hyperprolactinemic patients. Gibson *et al.* study, which included non-diabetic obese patients, found dramatic weight loss in both cabergoline and placebo groups (29).

Previous studies demonstrated that prolactin could reduce the lipoprotein lipase activity in human white adipose tissues, which lead to increase in triglyceride (TG) levels (30). Other studies verified the improvement effect of bromocriptine on lipid profile of hyperprolactinemic patients (20). Since our patients did not show significant changes in weight, BMI, and WC, other mechanisms could be involved in the beneficial effects of cabergoline on glucose metabolism.

Patients enrolled in this study had good adherence, and except for one patient with resistant vertigo, none of the other patients in the cabergoline group experienced any major side effects. Four patients (33%) in the cabergoline group and two patients (18%) in the placebo group reported mild side effects (mild headache and gastrointestinal upset) in the first weeks of starting the treatment. All cabergoline-treated patients had remarkable decrease in prolactin level, which clearly demonstrated their adherence. 

Although there is a concern of increased risk of fibrotic and valvular heart disease in patients treated with ergot derivatives such as cabergoline, this serious adverse drug reaction does not occur at the low doses used to treat hyperprolactinemia. In addition, other investigations have confirmed the ease of use and acceptable profile of adverse drug reactions of cabergoline in long-term use (31,32) and favorability of cabergoline versus bromocriptine in the treatment of hyperprolactinemia due to its low adverse effect, and higher efficacy in normalization of prolactin (33). 

## Conclusion

Cabergoline is an acceptable biweekly medication versus once or twice daily bromocriptine for improving glucose metabolism homeostasis. Previous studies have shown that cabergoline improves glucose metabolism in impaired glucose metabolism disorders (IGT, IFG). The proposed mechanisms of dopamine agonists in glucose metabolism motivated us to hypothesize and design a pilot study to examine it. In the present study, FPG, 2-h-PG, 2-h insulin, HOMA2 %S, and HOMA2 IR showed significant improvement, and changes in other factors showed a positive trend. However, over 16 weeks these other changes were not significant, and clinical studies of longer duration could confirm these effects. The results were impressive and motivated us to design a larger clinical trial of longer duration involving more patients with impaired glucose metabolism to investigate this in greater detail. 
